# Development and implementation of the AIDA international registry for patients with Schnitzler's syndrome

**DOI:** 10.3389/fmed.2022.931189

**Published:** 2022-07-18

**Authors:** Jurgen Sota, Antonio Vitale, Ewa Więsik-Szewczyk, Micol Frassi, Giuseppe Lopalco, Giacomo Emmi, Marcello Govoni, Amato de Paulis, Achille Marino, Antonio Gidaro, Sara Monti, Daniela Opris-Belinski, Rosa Maria R. Pereira, Karina Jahnz-Rózyk, Carla Gaggiano, Francesca Crisafulli, Florenzo Iannone, Irene Mattioli, Francesca Ruffilli, Ilaria Mormile, Katarzyna Rybak, Valeria Caggiano, Paolo Airò, Abdurrahman Tufan, Stefano Gentileschi, Gaafar Ragab, Ibrahim A. Almaghlouth, Adham Aboul-Fotouh Khalil, Marco Cattalini, Francesco La Torre, Maria Tarsia, Henrique A. Mayrink Giardini, Moustafa Ali Saad, Monica Bocchia, Federico Caroni, Teresa Giani, Elisa Cinotti, Piero Ruscitti, Pietro Rubegni, Marília A. Dagostin, Bruno Frediani, Aslihan Avanoglu Guler, Francesca Della Casa, Maria Cristina Maggio, Andreas Recke, Dagmar von Bubnoff, Karoline Krause, Alberto Balistreri, Claudia Fabiani, Donato Rigante, Luca Cantarini

**Affiliations:** ^1^Research Center of Systemic Autoinflammatory Diseases and Behçet's Disease Clinic, Department of Medical Sciences, Surgery and Neurosciences, University of Siena, Siena, Italy; ^2^Department of Internal Medicine, Pulmonology, Allergy and Clinical Immunology, Central Clinical Hospital of the Ministry of National Defence, Military Institute of Medicine, Warsaw, Poland; ^3^Rheumatology and Clinical Immunology, Spedali Civili and Department of Clinical and Experimental Sciences, University of Brescia, Brescia, Italy; ^4^Rheumatology Unit, Department of Emergency and Organ Transplantation, University of Bari, Bari, Italy; ^5^Department of Experimental and Clinical Medicine, University of Florence, Florence, Italy; ^6^Rheumatology Unit, Department of Medical Sciences, Azienda Ospedaliero-Universitaria S. Anna - Ferrara, University of Ferrara, Ferrara, Italy; ^7^Section of Clinical Immunology, Department of Translational Medical Sciences, University of Naples Federico II, Naples, Italy; ^8^Department of Translational Medical Sciences, Center for Basic and Clinical Immunology Research (CISI), "World Allergy Organisation Center of Excellence, University of Naples Federico II, Naples, Italy; ^9^Unit of Pediatric Rheumatology, ASST Gaetano Pini-CTO, Milan, Italy; ^10^Department of Biomedical and Clinical Sciences Luigi Sacco, Luigi Sacco Hospital, University of Milan, Milan, Italy; ^11^Rheumatology Department, Istituto di ricovero e cura a carattere scientifico Policlinico S. Matteo Fondazione, University of Pavia, Pavia, Italy; ^12^Department of Internal Medicine and Rheumatology, Carol Davila University of Medicine and Pharmacy, Bucharest, Romania; ^13^Rheumatology Division, Hospital das Clinicas (HCFMUSP), Faculdade de Medicina, Universidade de São Paulo, São Paulo, Brazil; ^14^Department of Internal Medicine, Nephrology and Endocrinology, Clinical District Hospital No. 2, Rzeszów, Poland; ^15^Division of Rheumatology, Department of Internal Medicine, Gazi University Faculty of Medicine, Ankara, Turkey; ^16^Unit of Rheumatology, Azienda Ospedaliero-Universitaria Senese, Siena, Italy; ^17^Rheumatology and Clinical Immunology Unit, Internal Medicine Department, Faculty of Medicine, Cairo University, Giza, Egypt; ^18^Faculty of Medicine, Newgiza University (NGU), Giza, Egypt; ^19^Rheumatology Unit, Department of Medicine, College of Medicine, King Saud University, Riyadh, Saudi Arabia; ^20^College of Medicine Research Center, College of Medicine, King Saud University, Riyadh, Saudi Arabia; ^21^The Egyptian School for Musculoskeletal Ultrasonography (EgySMUS), Cairo, Egypt; ^22^Pediatric Clinic, University of Brescia and Spedali Civili di Brescia, Brescia, Italy; ^23^Clinical Pediatrics, University of Bari, Bari, Italy; ^24^Hematology, Azienda Ospedaliera Universitaria Senese, University of Siena, Siena, Italy; ^25^Unit of Dermatology, Department of Medical, Surgical and Neurological Sciences, University of Siena, Siena, Italy; ^26^Rheumatology Unit, Department of Biotechnological and Applied Clinical Sciences, University of L'Aquila, L'Aquila, Italy; ^27^University Department Pro.Sa.M.I. “G. D'Alessandro”, University of Palermo, Palermo, Italy; ^28^Department of Dermatology, Allergology and Venerology, University Hospital Schleswig-Holstein, Campus Lübeck, Lübeck, Germany; ^29^Department of Dermatology, Venerology and Allergology, Charité–Universitätsmedizin Berlin, Berlin, Germany; ^30^Bioengineering and Biomedical Data Science Lab, Department of Medical Biotechnologies, University of Siena, Siena, Italy; ^31^Ophthalmology Unit, Department of Medicine, Surgery and Neurosciences, University of Siena, Siena, Italy; ^32^Department of Life Sciences and Public Health, Fondazione Policlinico Universitario A. Gemelli IRCCS, Rome, Italy; ^33^Institute of Pediatrics, Rare Diseases and Periodic Fevers Research Centre, Università Cattolica Sacro Cuore, Rome, Italy

**Keywords:** autoinflammatory disease, rare disease, international registry, personalized medicine, biotherapies, interleukin-1

## Abstract

**Objective:**

The present paper describes the design, development, and implementation of the AutoInflammatory Disease Alliance (AIDA) International Registry specifically dedicated to patients with Schnitzler's syndrome.

**Methods:**

This is a clinical physician-driven, population- and electronic-based registry implemented for the retrospective and prospective collection of real-life data from patients with Schnitzler's syndrome; the registry is based on the Research Electronic Data Capture (REDCap) tool, which is designed to collect standardized information for clinical research, and has been realized to change over time according to future scientific acquisitions and potentially communicate with other existing or future similar registries.

**Results:**

Since its launch, 113 centers from 23 countries in 4 continents have been involved. Fifty-seven have already obtained the approval from their local Ethics Committees. The platform counts 324 users (114 Principal Investigators, 205 Site Investigators, 2 Lead Investigators, and 3 data managers) at current (April 28th, 2022). The registry collects baseline and follow-up data using 3,924 fields organized into 25 instruments, including patient's demographics, history, clinical manifestations and symptoms, trigger/risk factors, laboratory, instrumental exams, therapies, socioeconomic information, and healthcare access.

**Conclusions:**

This International Registry for patients with Schnitzler's syndrome facilitates standardized data collection, enabling international collaborative projects through data sharing and dissemination of knowledge; in turn, it will shed light into many blind spots characterizing this complex autoinflammatory disorder.

## Introduction

Despite being individually uncommon, rare diseases affect a significant proportion of the general population if taken collectively: they actually represent a huge burden to society in terms of direct and indirect social, economic and healthcare costs (1). In this context a considerable proportion of inpatient burden, hospital admissions, orphan drug sales and impact on community medicine is connected with the management of rare diseases, especially in the Western countries (2–4). Moreover, clinical trials in the field of rare diseases are difficult to conduct due to the low epidemiologic burden. Therefore, based on conventional recruitment methods, clinical trials are sparse and more likely single arm, non-randomized and open label studies (5). Taken together, these aspects highlight the substantial difficulties that physicians encounter in everyday clinical practice when dealing with rare diseases. In the context of autoinflammatory diseases, these limitations have generated the urgency to develop an international network capable of gathering together the Centers experienced with these conditions worldwide. In this regard, the AutoInflammatory Disease Alliance (AIDA) network has been developed with the aim to represent an international group of physicians and researchers interested in sharing their experience and information on the clinical, therapeutic and research approach to autoinflammatory diseases. This will facilitate a comprehensive description of disease manifestations, their long-term clinical course, prognostic outcomes and a targeted treatment approach tailored according to the patient's profile in the view of a personalized medicine.

The present paper has been proposed to describe the design, development and deployment of an international disease-specific registry specifically dedicated to Schnitzler's syndrome in the frame of the AIDA network. Schnitzler's syndrome is a very rare condition that has many similarities with the hereditary autoinflammatory diseases (6). Pathogenesis along with disease clinical course and prognosis are still far from being fully defined. Therefore, an international registry oriented to this very rare syndrome constitutes a precious source of data to be translated into valuable and solid evidence capable of significantly widening the current evidence on this disorder.

## Materials and Methods

### Study design

The Registry for Schnitzler's syndrome is classified as a clinical-, physician-driven, population- and electronic-based registry; it was developed along with other autoinflammatory disease-specific registries in the context of AIDA network (7–9).

Participation is open to any Center that deals with Schnitzler's syndrome regardless of location, medical specialty, or type of practice setting. Centers may join the AIDA network and obtain credentials to access the Registry after having officially requested an involvement into the Network to the study promoter. However, obtaining approval from the local Ethics Committee and appointing a Principal Investigator able to coordinate the study locally and at least one Site Investigator responsible for the documentation and data entry should be considered essential pre-requisites. As data collected refer to information routinely collected in the field of the best standard of care, there is neither cost nor financial compensation for the study participation.

Data collection is made up of a retrospective and a prospective phase: the former refers to the information routinely gathered during the past years of active disease up to the inclusion in the registry; the latter includes clinical, therapeutic, and socioeconomic information collected starting from the moment of enrolment. It is advisable to insert the retrospective data with the patient actively participating in the recruitment, in order to obtain as many details as possible, minimizing missing data and any recall bias. Regarding the prospective collection, data have to be updated at least annually or in case of treatment changes, as for additional therapy and/or posology adjustments.

According to its observational nature, the registry includes demographic, genetic, clinical, laboratory, diagnostic and therapeutic data; long-term outcomes and prognostic variables will also be collected if written informed consent will not be withdrawn over time. Neither the clinical management, nor the adherence to the study is influenced by the study participation in any way.

### Study objectives

The primary aim of this registry is to bypass the limitations related to the standard research approach and to remedy the poor number of patients available for each Center.

Other objectives consist in the following points: (I) to fully characterize the disease phenotype and its changes during the long-term follow-up; (II) to point out the prognosis of Schnitzler's syndrome in the light of the new therapeutic acquisitions; (III) to identify predictive variables to therapeutic response; (IV) to refine the process of differential diagnosis; (V) to analyze the role of posology adjustments when primary or secondary inefficacy occur; (VI) to study the behavior of Schnitzler's syndrome during pregnancy and post-partum period and possible therapeutic strategies to employ in pregnant or breastfeeding women; (VII) to estimate the socioeconomic impact of Schnitzler's syndrome and the benefits potentially obtained with therapy; (VIII) to comprehensively define hematological complications in the long-term; (IX) to report the cardiovascular complications in patients with Schnitzler's syndrome; (X) to develop recommendations useful for everyday clinical management.

As the enrolling process will expand, it would be possible to design more specific and cutting-edge studies according to future unmet needs.

### Ethical/legal aspects

The first national regulatory approval of the AIDA Project was obtained in June 2019 by the Ethics Committee of the Azienda Ospedaliero-Universitaria Senese, Siena, Italy (Ref. No. 14951). Later, national, and international expert centers for the diagnosis, clinical management and treatment of autoinflammatory diseases have approved the project before joining the AIDA network.

Patients' data are kept in accordance with the EU General Data Protection Regulations (GDPR) about the protection and processing of personal data (2016/679/EU) (10).

This project was registered at ClinicalTrials.gov (ID: NCT05200715) and follows the principles of the Declaration of Helsinki.

After having received age-appropriate information sheets, patients (or their parents/legal guardian) have to give their voluntary informed consent; minors aged ≥12 years are also required to provide their assent to be included in the study.

Patients have to properly receive information about aims of the study, terms of data collection and management, rules for data access, and possible withdrawal of the consent to continue data collection. In addition, both patients and Principal Investigators may withdraw their consent for the use of data for statistical analyses at any time. In this case, all gathered data will be deleted soon after the patient and/or Principal Investigator communication to the study promoter.

Patients will not receive any honoraria or other payments for the participation in this project. Also, no relationships to billing of the healthcare system or insurance companies have to be disclosed.

### Patients' eligibility

Inclusion criteria for the recruitment into this AIDA Registry consist in the fulfillment of Strasbourg diagnostic criteria for Schnitzler's syndrome (11). The lack of a written informed consent by the patient or her/his parents or legal guardian accounts for the only exclusion criterium considered in this project.

### Data collection

Data are collected through the Research Electronic Data Capture (REDCap), a metadata-driven software application and novel metadata-gathering workflow developed at Vanderbilt University Medical Center, routinely used to support translational research projects in the academic research environment (12).

Each Principal and Site Investigator included into the AIDA Project is provided with his/her own password and login identification to access the registry through the REDCap web-interface, insert data on the Instruments of the registry and then review or complete the already inserted information. None of the participating Principal and Site Investigators are allowed to check information uploaded from other Centers.

While the public website (https://aidanetwork.org/en/) may be accessible by anyone who wants to learn more about AIDA network, its objectives and how to participate to the project, the registry website (https://sitbio.med.unisi.it/redcap/redcap_v12.2.7/index.php?pid=28) is hosted on a separate password-protected platform.

Data are stored on a server placed in the University of Siena, Siena, Italy. Ownership of results generated from the analysis of aggregated data will belong to the Promoter.

### Statistical analysis

Data collected will be converted into an appropriate format for statistical analysis. To this end, a good and reliable statistical plan is warranted according with the specific objectives that will be pursued. The statistical plan will include general principles related to descriptive statistics as well as inferential statistics.

Statistical analysis will take into consideration missing data before performing computations. Given the real-life context of data collection, a threshold level of missing data is set to 25%.

## Results

### Current numbers and registry development

Since its launch on November 27th, 2020, the AIDA project has quickly reached a wide geographic coverage: 113 centers have already joined the project around the world (by April 28th, 2022).

**Figure 1:**
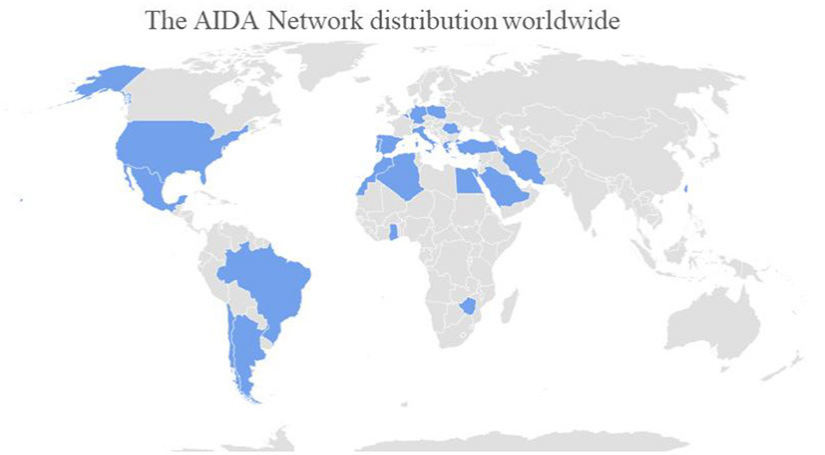
Countries involved in the AIDA Network (updated on April 28th, 2022).

To date, 3,924 common data elements (fields) organized into 25 instruments (forms) compose the registry. The full list of instruments and their fields are listed in Table 1. The fields for data collection are organized in such a way as to appear only if patient's clinical history make necessary to answer, according to a branching structure. Therefore, only a part of the 3,924 fields will initially appear to the investigators, and the number of questions to answer in the registry will depend exclusively on patient's clinical complexity. Table 2 provides the full list of the objectives of the registry for the future agenda. Figure 1 shows AIDA network distributions around the world.

**Table 1:** List of instruments (to be regarded as “forms”) included in the registry dedicated to patients with Schnitzler's syndrome, with the corresponding number of common data elements, time-points at which they should refer to and number of mandatory fields.

**Instruments**	**Fields**	**Retrospective/prospective phase**	**No. of mandatory fields**
Demographics	9	Retrospective phase	3
Consents	4	Retrospective/prospective phase	1
Diagnostic data and family history	20	Retrospective phase	2
Features of attacks at the time of disease onset	26	Retrospective phase	0
Features of attacks up to the time of diagnosis	44	Retrospective phase	0
Features of attacks from the diagnosis to the enrolment into the registry	44	Retrospective phase	0
Clinical diagnostic scores and criteria	14	Retrospective/prospective phase	0
Laboratory data	17	Retrospective phase	1
Cardiovascular risk	23	Retrospective phase	2
Past and current treatments	1	Retrospective phase	0
NSAIDs monotherapy—the retrospective phase	74	Retrospective phase	1
Corticosteroid monotherapy/main therapy—the retrospective phase	131	Retrospective phase	1
Antihystamines—the retrospective phase	12	Retrospective phase	0
Colchicine treatment—the retrospective phase	89	Retrospective phase	1
Treatment with cDMARDs (not associated to biotechnological agents)—the retrospective phase	387	Retrospective phase	6
Treatment with small molecules not associated to biologic agents—the retrospective phase	756	Retrospective phase	12
Treatment with biologic agents—the retrospective phase	1,022	Retrospective phase	14
Fertility and pregnancy	14	Retrospective/prospective phase	1
Disease course and treatment during pregnancies	66	Retrospective/prospective phase	1
Follow-up visits—the prospective phase	647	Prospective phase	55

**Table 2:** Objectives of the registry dedicated to Schnitzler's syndrome in the platform of AIDA network.

Main objective	To bypass the limitations related to the standard research and to remedy the poor number of patients available for studies
Other objectives	To fully characterize the disease phenotype and its changes during follow-up
	To point out the prognosis of the syndrome
	To identify predictive variables for the therapeutic response
	To refine the process of differential diagnosis
	To analyze the role of posology adjustments in the case of drug inefficacy
	To study the disease course during pregnancy/post-partum period and eventual therapeutic strategies useful in pregnant or breastfeeding women
	To estimate the socioeconomic impact of the syndrome and the benefits obtained with therapy
	To define long-term hematological complications
	To report the cardiovascular complications
	To develop recommendations useful for routine clinical management

Data elements correspond to patient's demographics, medical history, laboratory features, symptoms at onset, symptoms developed over time, comorbidities, cardiovascular risk, work-up exams, pregnancies, long-term clinical outcomes, past and current treatments. On the other hand, longitudinal data are captured through a specific “follow-up” instrument, including disease activity, disease manifestations occurred in the last follow-up period, laboratory exams worn by the patient at the last assessment, any treatment adjustments, clinimetric scores, any hematologic complications, socio-economic details about access to the healthcare system, absenteeism and working capacity.

### Patients' involvement as key stakeholders

In recent years patients have become increasingly aware of their role, which is central in stimulating the research efforts and quality of clinical management. Patient advocacy groups may help by disseminating information, supporting the recruitment of patients, and taking part in regulatory processes. At current, many different associations have already taken part into the AIDA project, as for the Italian Association of Periodic Fevers (A.I.F.P., *Associazione Italiana Febbri Periodiche*), the National Association for Rheumatologic and Rare Diseases (A.P.M.A.R.R., *Associazione Nazionale Persone con Malattie Reumatologiche e Rare*), and the National Rheumatic Diseases Association (A.N.M.A.R., *Associazione Nazionale Malati Reumatici*). The involvement of patients' associations in other countries is actively ongoing to include a higher number of proactive components in the AIDA project.

## Discussion

Thanks to the new technologies and online tools for the worldwide sharing of information, recent years have witnessed a rapid proliferation of rare diseases registries, which have completely changed the approach to research and participation in international projects. Indeed, according to Orphanet, a European website aimed at providing information about orphan drugs and rare diseases, there are more than 793 current European registries dedicated to rare diseases (13). Their goals are heterogeneous and range from clinical management to epidemiology and research projects; each of them is supported by a wide variety of information systems, data collection and management tools. In this context, the AIDA network has been developed with the aim of overcoming the current issues in the field of rare autoinflammatory diseases, including the fragmentation of knowledge and research affecting Schnitzler's syndrome. This condition is considered as the paradigm of late-onset acquired autoinflammatory syndromes (6). Despite this, the diagnostic delay often accompanied with several misdiagnosis especially during the initial phases, is already remarkable (roughly 5 years), complicating the disease course with significant morbidity due to the lack of a proper treatment. The advent of IL-1 blocking agents on the other hand has dramatically changed the management of Schnitzler's syndrome (14), which are now considered as a first-line therapy. This leads to a considerable health burden with a notable decrease in the quality of life and an even greater impact on the healthcare system. The low incidence and prevalence of Schnitzler's syndrome not only results in a reduced awareness of this condition—and thus the ability to include it in differential diagnosis—but makes it a clinical and therapeutic conundrum. Therefore, an increased awareness is needed among physicians to improve patients' quality of life and enhance the overall prognosis of this disease, which is still largely dependent on the onset of hematological complications (15).

The development and activation of this international registry represents an invaluable opportunity to widen the knowledge about this unusual disease through the accrual of real-life data, obtaining solid scientific information and final real-world evidence to apply in the everyday medical practice. Its pioneer mission is to create an international network of researchers capable of joining forces for the common purpose to solve the unmet needs that will gradually arise for both patients and physicians.

In this regard, the AIDA network has joined together the different specialties involved in the management of Schnitzler's syndrome, including rheumatologists, dermatologists, immunologists, hematologists, internal medical physicians, and radiologists. These figures may now communicate with each other globally, resulting in the final goal to create an effective strategic approach toward challenges associated with this rare disease.

Among other things, this Registry will clarify the geographical distribution of the disease and any change in clinical expression according to the environmental contexts. Also, interesting areas of research would be to fully describe the range of disease manifestations and their change over time; to improve the diagnostic process in the field of systemic inflammatory diseases; to assess the prognosis in the light of the new treatment strategies; to understand which patients are more responsive to a specific therapy; to disclose the proper therapeutic approach when a first biologic line fails; to evaluate how therapy may improve the impact of the disease from a socioeconomic perspective.

The flexibility of the registry allows a rapid implementation of the tool in case of protocol variations. In this regard, the registry has the ability to change with the aim of also meeting future challenges arising from either new scientific acquisition or everyday clinical practice. In addition, the registry has the ability to communicate with any other present or future registry focused on the same disease.

The AIDA Registry for Schnitzler's syndrome shows the typical shortcomings of observational studies. In particular, entering data requires time and attention, especially when the patient's medical history is long or complex in terms of treatment attempts and number of disease complications; in this case, a high frequency of missing data could affect the research potentialities. Furthermore, investigators are not required to consecutively enroll patients followed in their Centers. This could lead to selection biases. Despite these limitations, this registry has the potential to definitively overcome the issues typically associated with the low epidemiological burden and poor number of patients to enroll in clinical trials. The real-life context of data collected in this registry will lead to the achievement of real-life evidence directly applicable in the care of patients with Schnitzler's syndrome. In conclusion, the development of the AIDA International Registry for patients with Schnitzler's syndrome will allow the collection of standardized information, enabling international multicentre collaborative research through data sharing and implementation and optimisation of scientific efforts worldwide.

## Ethics Statement

The studies involving human participants were reviewed and approved by Ethics Committee of the Azienda Ospedaliero-Universitaria Senese, Siena, Italy (Ref. No. 14951). Written informed consent to participate in this study was provided by the participants' legal guardian/next of kin.

## Author Contributions

JS wrote the first draft of the manuscript. AV conceived and designed the study and revised the draft of the manuscript. EW-S, MF, GL, GE, MG, AP, AM, AG, SM, DO-B, RP, KJ-R, CG, FCr, FI, IMa, FR, IMo, KR, VC, and PA were involved in data recruitment in the Registry dedicated to patients with Schnitzler's syndrome. AT, SG, GR, IA, AA-FK, MC, FT, MT, HG, MS, MB, FCa, EC, PRus, PRub, MD, BF, AG, FDC, MM, and CF were included in the authorship as investigators from to the top three contributor centers for any of the other AIDA Registries. AR, DB, and KK were included as leading AIDA experts in the field of Schnitzler's syndrome. AB is the bioengineer involved in the technical management of the platform and registries. DR took care of the final revision of the manuscript. LC conceived and designed the study and accounted for AIDA Registries Co-ordinator. Authorship has been established based on the number of data recruited in the AIDA Registries on April 20th, 2022. All authors contributed to the article and approved the submitted version. '

## Conflict of interest

The authors declare that the research was conducted in the absence of any commercial or financial relationships that could be construed as a potential conflict of interest.

## Publisher's Note

All claims expressed in this article are solely those of the authors and do not necessarily represent those of their affiliated organizations, or those of the publisher, the editors and the reviewers. Any product that may be evaluated in this article, or claim that may be made by its manufacturer, is not guaranteed or endorsed by the publisher.
